# Field homogeneity improvement of maglev NdFeB magnetic rails from joints

**DOI:** 10.1186/s40064-016-1965-3

**Published:** 2016-03-25

**Authors:** Y. J. Li, Q. Dai, C. Y. Deng, R. X. Sun, J. Zheng, Z. Chen, Y. Sun, H. Wang, Z. D. Yuan, C. Fang, Z. G. Deng

**Affiliations:** Applied Superconductivity Laboratory, State Key Laboratory of Traction Power, Southwest Jiaotong University, Chengdu, 610031 People’s Republic of China; School of Information Science and Technology, Southwest Jiaotong University, Chengdu, 610031 People’s Republic of China; School of Mechanical Engineering, Southwest Jiaotong University, Chengdu, 610031 People’s Republic of China; School of Electrical Engineering, Southwest Jiaotong University, Chengdu, 610031 People’s Republic of China

**Keywords:** Magnetic rail, Joint, Field homogeneity, Maglev

## Abstract

An ideal magnetic rail should provide a homogeneous magnetic field along the longitudinal direction to guarantee the reliable friction-free operation of high temperature superconducting (HTS) maglev vehicles. But in reality, magnetic field inhomogeneity may occur due to lots of reasons; the joint gap is the most direct one. Joint gaps inevitably exist between adjacent segments and influence the longitudinal magnetic field homogeneity above the rail since any magnetic rails are consisting of many permanent magnet segments. To improve the running performance of maglev systems, two new rail joints are proposed based on the normal rail joint, which are named as mitered rail joint and overlapped rail joint. It is found that the overlapped rail joint has a better effect to provide a competitive homogeneous magnetic field. And the further structure optimization has been done to ensure maglev vehicle operation as stable as possible when passing through those joint gaps. The results show that the overlapped rail joint with optimal parameters can significantly reduce the magnetic field inhomogeneity comparing with the other two rail joints. In addition, an appropriate gap was suggested when balancing the thermal expansion of magnets and homogenous magnetic field, which is considered valuable references for the future design of the magnetic rails.

## Background

In 2014, a new generation of the high temperature superconducting (HTS) maglev indoor experiment platform in enclosed tubes has been successfully developed in Southwest Jiaotong University. It is composed of a HTS maglev vehicle named as Super-Maglev, evacuated tubes whose vacuum degree is adjustable, and a circular Halbach-type magnetic rail. The HTS maglev has the development potential in the area of urban and cargo transportation for its advantages of high speed, low noise, riding comfort and safety (Ma et al. 2003).

Magnetic rail is one of the core components in the HTS maglev system, which is used to provide magnetic field source to the whole levitation system. By employing the Halbach-type magnetic rail (Jing et al. 2007; Deng et al. 2009), a strong magnetic field with high gradient in distribution can be achieved for providing stable suspension (Wang et al. 2001; Sotelo et al. 2011; Deng et al. 2015). Magnetic field is particularly required to be as homogeneous as possible along the running direction, so that a friction-free movement can be achieved in theory. In fact, the longitudinal magnetic field above the magnetic rail is usually not sufficiently homogeneous as excepted for the practical applications, the main obstacle is that the long distance rails are assembled by lots of short rail segments (Okano et al. 2004). There are joint gaps between every adjacent segment. Those joint gaps are the major factor affecting the magnetic field inhomogeneity. The inhomogeneous magnetic field has been verified as a kind of external disturbance for the maglev vehicle, and is a non-ignorable factor that directly affects the riding comfort and the running safety (Lin et al. 2011; Sun et al. 2016).

Enormous work has been done to explore the influence of magnetic field inhomogeneity due to joint gaps. Okano et al. (2004) and Lin et al. (2011) calculated the magnetic field inhomogeneity between two segments of the conventional unimodal magnetic rail and put forward some improvement countermeasures. The cost of the Halbach-type magnetic rail is just about 38 % of that of conventional unimodal magnetic rail per kilometer (Wang et al. 2002 and Del-Valle et al. 2011), while the levitation efficiency (N/cm^3^) is about 2.85 times larger (Deng et al. 2008), and thus has been widely used or reformed for the maglev applications (Guo et al. 2010; Deng et al. 2013; Boughrara et al. 2013). But until recently, the method to improve magnetic field homogeneity of the Halbach-type magnetic rail with joint gaps is so limited that it is necessary to optimize the Halbach-type magnetic rail joint to guarantee the riding comfort of the HTS maglev vehicle. In this paper, the magnetic field inhomogeneity over normal rail joint which was employed in the present Maglev system was evaluated. Two rail joints, the mitered rail joint and the overlapped rail joint, have been proposed aiming at improving the longitudinal homogeneity of the applied magnetic fields. The simulation results show the effectiveness of the new rail joints, especially for the overlapped rail joint, and the further optimized rail joint has better performance.

## Model building

**Fig. 1:**
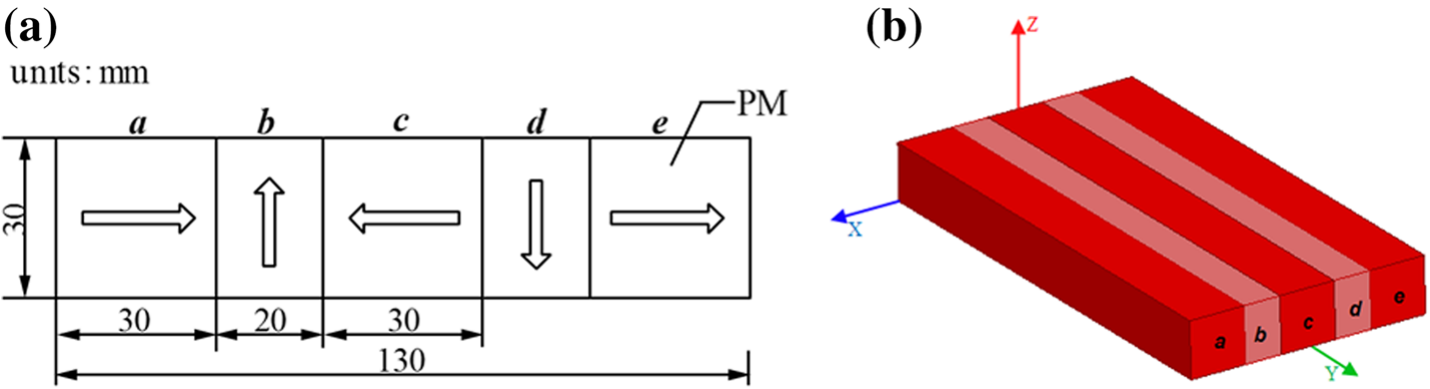
**a** Cross-section view of the Halbach magnetic rail, arrows depict the polarity of PMs; **b** computational 3D model of the magnetic rail

Halbach array is a creative arrangement type of permanent magnet (PM) array. Figure 1a shows the cross-section view and the size of the Halbach-type magnetic rail for the HTS maglev system is 200 × 130 × 30 mm^3^. The arrows represent the magnetization directions of the PMs. The PMs were orderly labeled as *a*, *b*, *c*, *d* and *e* from left to right as shown in Fig. 1.

**Fig. 2:**
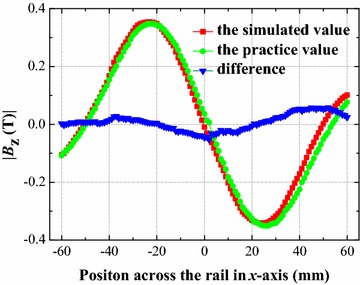
Comparison of simulated and practice values at the levitation height of 15 mm above the rail, profile along the cross section of the rail

A Halbach–type magnetic rail segment was built by the ANSOFT MAXWELL software. All models were built in 3D, in which the applied permanent magnet material NdFeB was N45. The specific parameters were set as below: remanence (*B*_r_) is 1.36 T; coercivity force (*H*_c_) is 994 kA/m; and the relative permeability (*μ*) is 1.09977854. As shown in Fig. 2, the measured and simulated results are in good agreement, which prove the validity of the finite element analysis.

**Fig. 3:**
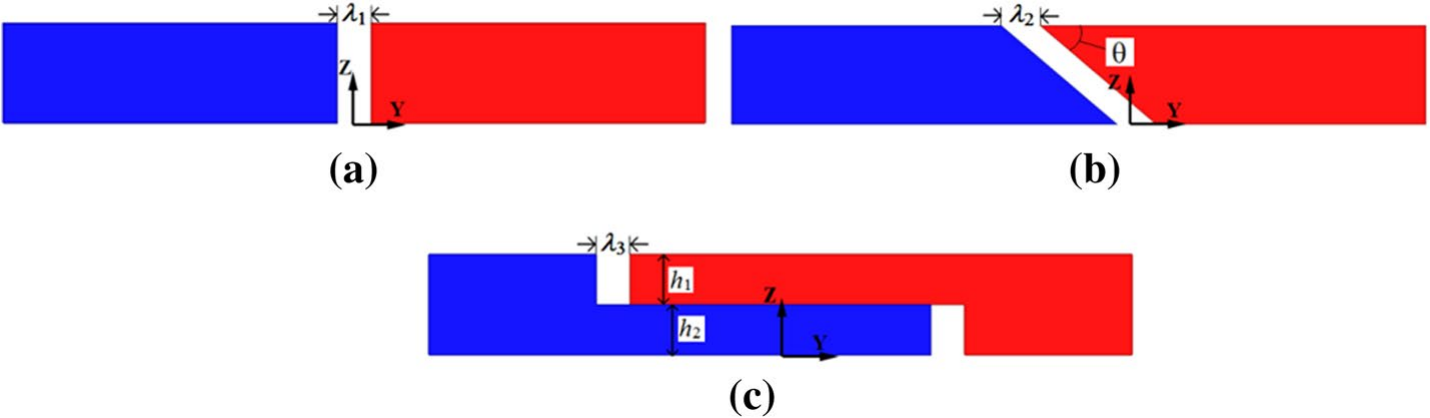
Side view of three rail joints: **a** normal rail joint (RJ I) whose joint gap is a coherent cuboid; **b** mitered rail joint (RJ II) whose joint gap is a parallelepiped; **c** overlapped rail joint (RJ III)whose joint gaps are two incoherent cuboids

Figure 3a displays a normal rail joint (RJ I) with a cuboid joint gap. It is found that this kind of joint gap will lead to magnetic field inhomogeneity. Two rail joints were proposed to improve the magnetic field homogeneity. One of them is a mitered rail joint (RJ II). Its side view with a joint gap is shown in Fig. 3b. In this structure, the left and right magnetic segments meet in a miter joint; hence the shape of the joint gap between two segments is a parallelepiped. The other one is an overlapped rail joint (RJ III), as presented in Fig. 3c, the rail joint include two layers, and each layer is 100 mm in length with an un-continuous cuboid shape. In order to have a series of comparable results, the models were set in the same total length of 200 mm.

Coordinate systems of these three rails were set up as shown in Fig. 3. The origins of the coordinate system are established on the bottom of the geometric center of the gaps. *X*-axis is perpendicular to the running direction and *Y*-axis parallels to the running direction of the magnetic rail and its direction is from left to right. *Z*-axis is along the vertical direction.

## Feasibility study

The magnetic field density *B*_x_ and *B*_z_ decrease with the increase of the joint gap. In order to clearly describe the change of magnetic field distribution, we define these changes in ratio as below:

$$ \begin{aligned} \eta_{\text{x}} & = \left( {\left| {B_{{{\text{x}}\_{\text{dev}}}} } \right| - \left| {B_{{x\_{\text{nor}}}} } \right|} \right)/B_{{{\text{x}}\_{\text{nor}}}} \times 100\,\% \\ \eta_{\text{z}} & = \left( {\left| {B_{{{\text{z}}\_{\text{dev}}}} } \right| - \left| {B_{{{\text{z}}\_{\text{nor}}}} } \right|} \right)/B_{{{\text{z}}\_{\text{nor}}}} \times 100\,\% \\ \end{aligned} $$

where $\left| {B_{{{\text{x}}\_{\text{nor}}}} } \right|$ is the maximum value of lateral component of magnetic field without any defects, $\left| {B_{{{\text{x}}\_{\text{dev}}}} } \right|$ is the maximum value of lateral component of magnetic field with certain joint gap. These parameters are used to determine the influence of the joint gap on magnetic field. In practical application, the working height of the HTS maglev vehicle is about 15 mm. For this reason, we focus on the magnetic field distributions at the working height of 15 mm.

### Magnetic field of the normal rail joint

**Fig. 4:**
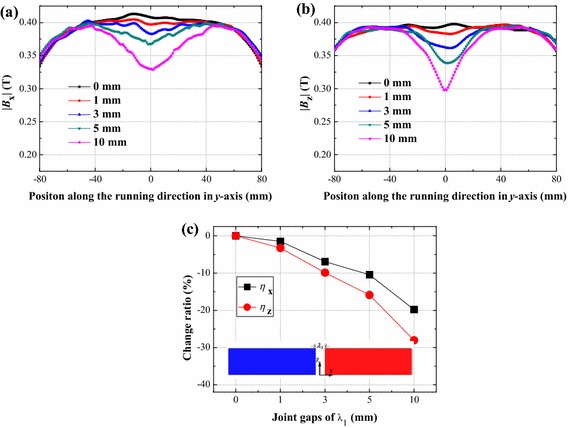
Influence of the joint gap (*λ*_1_) of the normal rail joint on **a** |*B*_x_| and **b** |*B*_z_| profile along the longitudinal direction of the rail; **c** change ratios of |*B*_x_| and |*B*_z_| with different joint gaps

Figure 4 displays the calculation results for components of magnetic field |*B*_x_| (a) and |*B*_z_| (b) of the normal rail joint with joint gaps (*λ*_1_) of 0, 1, 3, 5 and 10 mm. |*B*_x_| was plotted in *y*–*z* plane above the middle of magnet *c*, and |*B*_z_| was calculated in *y*–*z* plane above the middle of magnet *b*. In the case of a small joint gap of 1 mm, magnetic field hardly changes. Whereas a joint gap of 10 mm results in a large change of |*B*_x_| and |*B*_z_|. Figure 4c shows magnetic field change ratio of |*B*_x_| and |*B*_z_|. When *λ*_1_ = 10 mm, change ratio of |*B*_z_| is about −28.9 %, and change ratio of |*B*_x_| is about −19.4 %. The decrease of |*B*_z_| will lead to the decrease of the levitation force of HTS maglev vehicle systems. When the onboard superconductors pass through the joint gap, vibration may happen on the vehicle.

### Comparison on magnetic fields of three different rail joints

From the above analysis, it points out that the magnetic field above the normal rail joint is not homogeneous, and this magnetic field inhomogeneity will affect running performance of the levitated vehicle. Therefore, the mitered rail joint and the overlapped rail joint are put forward to improve the magnetic field to ensure the vehicle operate stable in a comparatively homogeneous magnetic field, as shown in Fig. 3b, c, respectively.

**Fig. 5:**
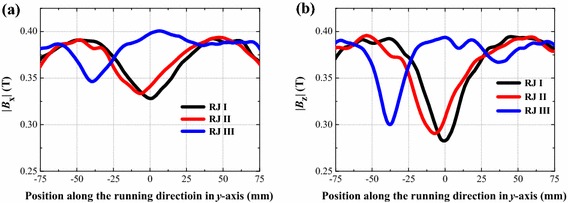
Comparisons on components of magnetic field of three rail joints with a 10 mm joint gap: **a** |*B*_x_| and **b** |*B*_z_|, profile along the longitudinal direction of the rail

Figure 5 respectively shows the magnetic field curves of |*B*_x_| and |*B*_z_| along the *y*-axis of three rail joints with a 10 mm joint gap (*λ*_1_ = *λ*_2_ = *λ*_3_ = 10 mm). The angle of *θ* is 45° in the mitered rail joint (RJ II). In the overlapped rail joint (RJ III), the heights of *h*_1_ and *h*_2_ are both 15 mm and the ratio of *h*_1_ to *h*_2_ is 1:1. Magnetic field distributions demonstrate that, with a 10 mm joint gap between two segments, magnetic field above the mitered rail joint and the overlapped rail joint are more homogeneous than that of the normal rail joint. Furthermore, the magnetic field distortion above the overlapped rail joint is the smallest.

## Structure optimization

Realizing an appropriate structure parameter is a significant way to improve homogeneity of magnetic field above a rail with certain joint gap. In order to improve the magnetic field as homogeneous as possible, structure optimizations of the mitered rail joint and the overlapped rail joint are further explored.

### The mitered rail joint

In Fig. 3b, it is seen that rail structure varies with the change of *θ*. Therefore, taking rail structure into account, magnetic field distributions above mitered rail joint with *θ* of 15°, 30°, 45°, 60° and 75° have been discussed.

**Fig. 6:**
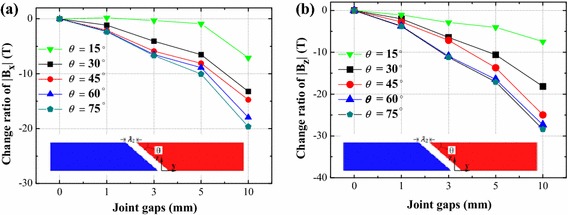
Change ratios of components of magnetic field **a** |*B*_x_| and **b** |*B*_z_| of the mitered rail joint with the same joint gaps

Figure 6 shows magnetic field change ratio of |*B*_x_| and *|B*_z_| according to different *λ*_2_ of 0, 1, 3, 5 and 10 mm. The curves prove that with the decrease of *θ* the magnetic field inhomogeneity decreases, when *θ* is 15°, magnetic field change ratio is the minimum in these five rail structure. With this structure, when the joint gap is 10 mm, the |*B*_x_| decreases by 0.0018 T (change ratio is −7.1 %), the |*B*_z_| decreases by 0.0674 T (change ratio is −7.65 %). Compared with magnetic field change ratio of the normal rail joint, we can conclude that the mitered rail joint is able to improve the magnetic field inhomogeneity to some extent. But when the *θ* reduce continuously, this kind of magnets will be difficult to fabricate. For this reason, a mitered rail joint with an angle of 30° is suggested, when *θ* is 30°, change ratio of |*B*_x_| and |*B*_z_| is −13.22 and −18.17 %, respectively.

### The overlapped rail joint

#### The Overlapped height

**Fig. 7:**
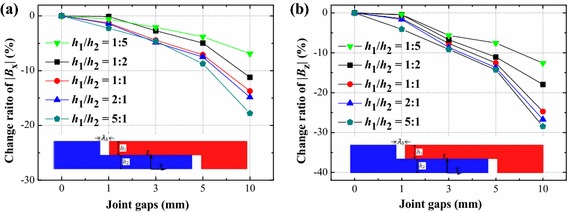
Change ratios of components of magnetic field **a** |*B*_x_| and **b** |*B*_z_| of overlapped rail joint with joint gaps

As well as the mitered rail joint, the overlapped rail structure changes with the height ratio *h*_1_/*h*_2_. If a same size joint gap occurs between two segments, magnetic field distribution varies with different rail structures. The magnetic field distributions above the overlapped rail joint with the ratio *h*_1_/*h*_2_ of 1:5, 2:1, 1:1, 1:2 and 5:1 are analyzed, and the sum of *h*_1_ and *h*_2_ is 30 mm. In the overlapped rail joint, |*B*_x_| and |*B*_z_| are calculated for *λ*_3_ = 0, 1, 3, 5 and 10 mm. Shapes of joint gaps are incoherent cuboid, hence magnetic field will distortion above those two joint gaps. As shown in Fig. 3c, they are called as upper joint gap and bottom joint gap, respectively. The upper joint gap is the main influence factor on the magnetic field inhomogeneity, so the magnetic field density of |*B*_x_| and |*B*_z_| above the upper joint gap was calculated. The change ratios of |*B*_x_| and |*B*_z_ |are shown in Fig. 7. It indicates when the ratio *h*_1_/*h*_2_ is 1:5; a maglev vehicle system can achieve a comparatively homogeneous magnetic field over the joint gap. But this kind of magnets are also difficult to fabricate. For this reason, an overlapped rail joint with the ratio *h*_1_/*h*_2_ of 1:2 is suggested. In this proportion, when the joint gap reaches to 10 mm, the |*B*_x_| decreased by 0.0455 T (change ratio is about −11.21 %), the |*B*_z_| decreased by 0.0700 T (change ratio is about −17.98 %).

**Fig. 8:**
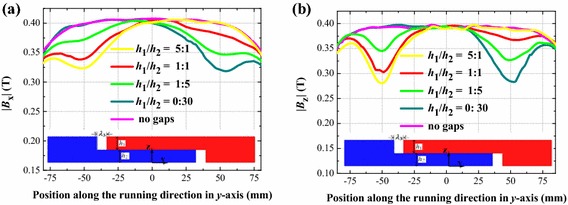
Magnetic field distributions **a** |*B*_x_| and **b** |*B*_z_| of the overlapped rail joint with a 10 mm joint gap with the ratio of *h*_1_/*h*_2_ changes, profile along the longitudinal direction of the rail

Curves shown in Fig. 8 represent the relationship between magnetic field distribution above the upper joint gap and bottom joint gap when the ratio of *h*_1_/*h*_2_ changes. It indicates that, when the ratio of *h*_1_/*h*_2_ becomes smaller, the magnetic field above the upper joint gap increases, while the magnetic field above the bottom joint gap decreases. When *h*_1_ decreases to 0 mm, and *h*_2_ increases to 30 mm, the overlapped rail joint turns into a normal rail joint. The magnetic field distribution at this situation was also plotted in Fig. 8, and the curve was labeled as 0:30. In the figure, magnetic field component |*B*_x_| was calculated in *y*–*z* plane above the middle of magnet *c*, |*B*_z_| was calculated in *y*–*z* plane above the middle of magnet *b*, and joint gap *λ*_3_ was set to 10 mm. Table 1 lists the change ratio above two incoherent cuboid joint gaps in different rail structures.

**Table 1:** Change ratio of overlapped rail joint with different rail structures

Proportion	Upper joint gap (%)		Bottom joint gap (%)	
	|*B*_x_|	|*B*_z_|	|*B*_x_|	|*B*_z_|
5:1	−17.80	−28.74	−1.46	−2.68
2:1	−14.85	−26.72	−2.70	−3.04
1:1	−13.72	−24.72	−4.54	−5.26
1:2	−11.21	−17.98	−6.5	−7.95
1:5	−6.88	−12.57	−13.21	−17.86
0:30	0	0	−19.4	−28.9

#### The lapped length

**Fig. 9:**
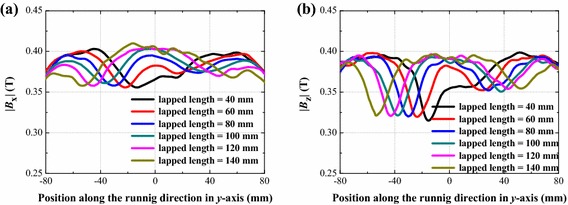
Components of magnetic field |*B*_x_| (**a**) and |*B*_z_| (**b**) of the overlapped rail joint with different lapped lengths

Figure 9 shows the magnetic field profiles of the overlapped rail joint with different lapped lengths. |*B*_x_| and |*B*_z_| were calculated for *λ*_3_ = 10 mm. When the lapped length decreases from 100 mm to 40 mm, the |*B*_x_| decreased by 0.0050 T (the change ratio is about −1.55 %), the |*B*_z_| decreased by 0.0063 T (change ratio is about −2 %). But when lapped length increases to 120 and 140 mm, the magnetic field become slightly inhomogeneous than the overlapped rail joint with a 100 mm lapped length. This is probably because of the limitation on the length of the simulation rail. In this paper, the simulation rails were all set to be 200 mm. When the lapped length becomes longer; the un-lapped length becomes shorter. In the practical application, when considering the manufacture ability of PM bars, we suppose 100 mm lapped length a good choice.

## Suitable size of the joint gap

PMs have the basic property of thermal expansion at high temperature, which may lead to the deformation and influence the operation performance of the maglev vehicle. Thus it is necessary to maintain a suitable size of the joint gap. In order to determine the most suitable size of the joint gap between two magnetic rail segments, the thermal expansion size of a magnetic rail was calculated. The coefficient of NdFeB’s thermal expansion is 4 × 10^−6^/°C. The formula of linear expansion is *α* = △*L*/*L* × △*T*, where *α* refers to coefficient of thermal expansion, △*L* length change of the object, △*T* temperature variation, *L* initial length of the object. Through this formula, with 35° temperature change (the common temperature variation from winter to summer in Chengdu), a Halbach rail of 1 meter long will increase 0.14 mm in summer compared to that in winter. Based on the above analysis and the magnetic field change ratio, it is suitable to have a joint gap around 1 mm.

Table 2 lists magnetic field change ratio of three rail joints with a 1 mm joint gap, *θ* of the mitered rail joint is 30° and ratio *h*_1_/*h*_2_ of the overlapped rail joint is 1:2. The data proves that magnetic field changes are very small and hardly influence maglev vehicle running performance.

**Table 2:** Change ratio of different rail joints with a 1 mm joint gap

	Normal rail joint		Mitered rail joint (*θ* = 30°)		Overlapped rail joint (*h*_1_:*h*_2_ = 1:2)	
	*|B*_x_|	*|B*_z_|	*|B*_x_|	*|B*_z_|	*|B*_x_|	*|B*_z_|
Change ratio	−1.50 %	−3.27 %	−1.15 %	−2.01 %	−0.1 %	−0.44 %

## Conclusion

To ensure the best performance of the maglev vehicle running above a long distance magnetic rail, two new rail joints, mitered rail joint and overlapped rail joint, have been put forward to obtain a comparatively homogeneous magnetic field, and structure parameter optimization was conducted. The simulation results show that mitered rail joint and overlapped rail joint are possible to obtain a comparatively homogeneous magnetic field than normal rail joint; the reason is that the rail with special joint shape always has magnets to compensate the magnetic field intensity above the joint gaps. The further optimizations show that overlapped rail joint can significant improve performance of the maglev vehicle when the ratio *h*_1_/*h*_2_ is 1:2. Considering the thermal expansion of magnets, the joint gap within 1 mm is suggested. The overlapped rail joint with optimized parameter does improve the performance of HTS Maglev system.

For further study, a small-size overlapped rail joint construction is planned to verify the function in improving the field homogeneity.
